# Controlling the Surface Morphology of Strongly Confined CsPbBr_3_ Perovskite Quantum Dots

**DOI:** 10.1021/acs.nanolett.6c01367

**Published:** 2026-07-15

**Authors:** Matthew L. Atteberry, Chance Lander, Carly Wickizer, Amila Wanasinghe, Chenjia Mi, Novruz G. Akhmedov, Sisi Xiang, Gavin C. Gee, Heath Hadley, Parker H. Bryan, John W. Peters, Madalina Furis, Yihan Shao, Yitong Dong

**Affiliations:** † Department of Chemistry and Biochemistry, 6187 The University of Oklahoma, Norman, Oklahoma 73019, United States; ‡ Materials Characterization Facility, 14736 Texas A&M University, College Station, Texas 77843, United States; § School of Materials Science and Engineering, 6187 The University of Oklahoma, Norman, Oklahoma 73019, United States; ∥ Homer L. Dodge Department of Physics and Astronomy, 6187 The University of Oklahoma, Norman, Oklahoma 73019, United States; ⊥ Center for Quantum Research and Technology, 6187 The University of Oklahoma, Norman, Oklahoma 73019, United States; # Materials Science and Engineering Program, 6187 The University of Oklahoma, Norman, Oklahoma 73019, United States

**Keywords:** Perovskite Nanocrystals, Morphology Control, Thermodynamic Equilibrium, Surface Ligands

## Abstract

Understanding the relationship between the structural and optical properties of lead halide perovskite quantum dots (QDs) requires precise control over their composition, size, surface passivation, and morphology. Although significant progress has been made in tuning the composition and size of perovskite QDs, less attention has been given to controlling their surface morphology, especially when the QDs are size-confined. Here, we present a synthesis of monodisperse, quantum-confined CsPbBr_3_ QDs with carefully controlled surface morphology. The exposure of surface facets is regulated by annealing the QDs with facet-selective dicationic ligands, which are designed to match the spacing of Cs^+^ vacancies on specific facets. Our QDs remain morphologically stable during purification and can self-assemble via drop-casting. By monitoring the circularity indices of QDs, we reveal that the evolution of surface facet exposure during annealing is driven thermodynamically. Our straightforward synthesis approach offers an additional degree of tuning freedom for perovskite QDs.

## Graphical abstract

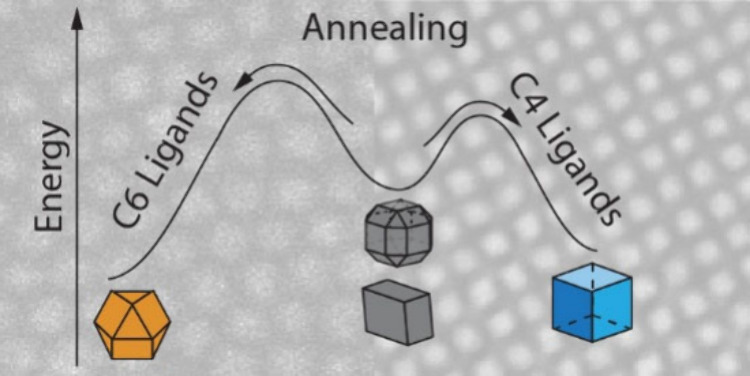

Colloidal lead halide perovskite quantum dots (QDs) have attracted significant attention due to their nearly perfect photoluminescence quantum yields, wide spectral tunability, and facile synthesis.^1,2^ Their outstanding optical properties have proven useful across a range of light-emitting technologies, including highly efficient LEDs, lasers, and quantum light sources.^3–8^ Additionally, perovskite QD assemblies have exhibited collective emission effects such as superfluorescence and superradiance.^9–11^ To fully understand how the perovskite QD structure affects their optical behavior and to control their self-assembly, precisely controlling the size and shape during synthesis is essential. Although the rapid growth rate and dynamic surface ligand binding to perovskite QDs pose challenges in producing highly uniform QDs,^12,13^ advances in synthetic control and regulation of QD growth mechanisms over the past decade have been made.^14–18^ To date, the size distribution and surface passivation of perovskite QDs have been greatly improved.^14,15,19,20^

While the size of perovskite QDs influences quantum confinement and multiexciton interactions, their shape also affects exciton degeneracy and surface chemical properties such as catalytic activity.^14,21–25^ Compared to the size control, less progress has been made in regulating the growth or transformation of surface facets.^26^ It has been demonstrated that controlling the dissolution of facets after QD formation can yield large perovskite nanocrystals with varying facet exposures.^27,28^ Depending on facet stability and ligands, polyhedral and multiarmed morphologies can be achieved.^29,30^ Additionally, exogenous metal cations and tertiary ammonium cations have been reported to yield CsPbBr_3_ nanocrystals with truncated cubic shapes.^31^ While these approaches mainly apply to large, weakly confined nanocrystals, directly regulating the surface facet exposure of strongly quantum confined perovskite QDs remains challenging. Successes have been achieved by regulating nucleation and growth kinetics and avoiding the use of alkylammonium cationic ligands. For example, using trioctylphosphine oxide, which forms complexes with Pb precursors, monodisperse small perovskite QDs can be formed in 30 min at room temperature.^14^ Employing anionic phosphonates, which strongly bind to Pb^2+^-terminated facets during QD synthesis, can also yield spherical QDs with sizes down to 5 nm.^32,33^ Despite achieving a decent size distribution through these low-temperature syntheses, studies and understanding targeting facet exposure control remain limited. It is also worth noting that postsynthetic purification can sometimes damage small QDs and alter their morphology, further complicating efforts to control both size and shape simultaneously.

The classical Gibbs–Curie–Wulff theorem states that a crystal’s shape depends on the relative surface energies of its facets.^34^ Although this argument was found less suitable for traditional CdSe QDs with slower growth kinetics, the low formation energy of perovskites may promote intraparticle ion migration, enabling thermodynamic tuning of perovskite QD shape.^35,36^ Here, we present the synthesis of highly confined, monodisperse CsPbBr_3_ perovskite QDs with precisely controlled surface morphologies through thermodynamic equilibrium. Diammonium cations have been reported as high-quality bidentate ligands to stabilize the surface of perovskite QDs.^37,38^ By annealing perovskite QDs with facet-selective dicationic ligands, we have produced cubical and polyhedral perovskite QDs with high uniformity throughout the ensemble. Using density functional theory (DFT) modeling, we find that when the distance between ammonium cationic binding groups matches the spacing of A-site cation vacancies on a specific facet, the surface energy is minimized. Annealing with these facet-selective ligands stabilizes the corresponding facets on QDs, leading to shapes that are thermodynamically favored. The synthesized perovskite QDs maintain their shape after purification and can self-assemble without controlling the drying rate under ambient conditions. The evolution of the perovskite QDs circularity indices during annealing illustrates facet evolution, supporting thermodynamic regulation of QD morphology. Our study offers insights into achieving full control over perovskite QD synthesis and producing highly uniform ensembles for superstructure fabrication, addressing experimental challenges in studying exciton-surface facet interactions.

High-temperature synthesis with alkylammonium halides often yields cubical perovskite QDs that tend to expose (100) facets.^39^ To produce multifaceted polyhedral QDs, (110) and (111) facets need to be exposed. Cationic ligands can fill the Cs^+^ vacancies on the perovskite surface and lead to a ligand-halide surface termination. Such a surface termination stabilizes fully formed [PbX_6_]^4–^ octahedra and can enhance PLQY and QD stability.^40^ However, the relatively small binding affinity of single alkylammonium halides, did not sufficiently stabilize the surface facets, and small QDs often exhibit oblate shapes with truncated corners.^41^ To enhance the ligand binding affinity of cationic ligands, dicationic molecules with two ammonium cations linked by a carbon chain have been used on weakly confined perovskite nanocrystals.^37,38^ The tunable linker offers an additional degree of freedom to adjust the geometric matching between binding moieties and surface vacancy patterns on different facets. For example, the neighboring Cs^+^ vacancies on (100) facets are ∼5.9 Å apart. On the (110) and the (111) facets, such an inter-Cs^+^ vacancy distance increases to approximately 8.5 Å (Figure 1). To bind both cationic centers in the ligand to a specific facet, the linker length must match the distance between two Cs^+^ vacancies. Note that although CsPbBr_3_ QDs can exhibit an orthorhombic or cubic phase, for simplicity, we have adopted the cubic-phase indices to represent the facets and crystal planes, given the very small *d*-spacing differences and similar atomic patterns.^22^

**Figure 1:**
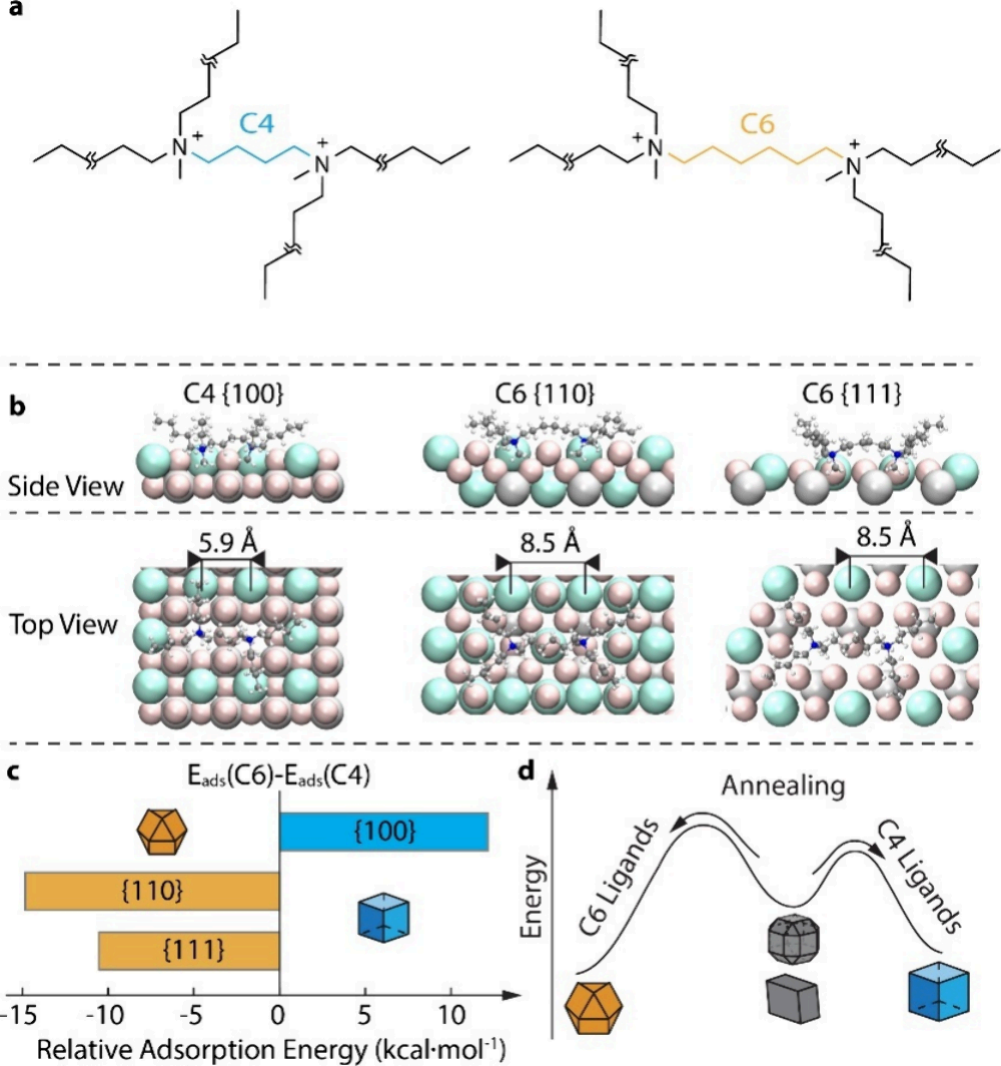
(a) Line structures of C4 and C6. (b) Top and side views of the (100) with C4 ligand bound and (110)/(111) with a C6 ligand bound. All facets are CsBr-terminated, and the ligands bind to Cs-vacancies. (c) Relative (to C4 ligands) surface adsorption energy of C6 ligands on the three facets in (a). Cyan, gray, and magenta spheres represent Cs^+^, Pb^2+^, and Br^–^ ions, respectively. (d) A thermodynamic diagram that illustrates the QD morphology regulation using geometric-matching ligands.

To understand how ligand-facet geometry compatibility influences the binding affinities of dicationic ligands on various facets, density functional theory (DFT)-based modeling was performed for the (100), (110), and (111) facets, each with a pair of neighboring Cs^+^ vacancies. Ligands with linkers containing 4 CH_2_ units (C4, 1,4-bis­(N-didodecyl-N,N-methylammonium)­butane dibromide) and 6 CH_2_ units (C6, 1,6-bis­(N-didodecyl-N,N-methylammonium)­hexane dibromide), which separate the ammonium cations by approximately 6.1 Å and 8.2 Å, respectively, are studied since the length of the linker closely matches the vacancy spacing on facets mentioned above (Figure 1a). Ligand tails, which often consist of long-chain aliphatic groups, are truncated to lower computational costs. Details of the DFT modeling are given in Supporting Information Note 1. Figure 1b shows the geometrically matched ligand-facet combinations: C4 on (100) and C6 on (110) and (111). In comparison, the ligand adsorption energies of mismatched ligand-facet combinations are also calculated (Supporting Information Table S2 and Figure S1). The difference in adsorption energy of the C4 and C6 on the three facets is plotted in Figure 1c to highlight the effects of ligand binding geometries. When the length of the ligand linker matches the vacancy spacing, the binding energy can improve by 10–15 kcal/mol. Closer examination of the bonded ligands shows that the C4 linker allows the two methyl groups on the ammonium cations to insert into two neighboring Cs^+^ vacancies on the (100) facet. Conversely, on the (110) and (111) facets, the C6 ligands fit better than the C4 ligands due to their longer linker. Meanwhile, the C4 linker is too short to enable bidentate binding, leaving one Cs^+^ vacancy unfilled. Note that in our model, the (111) facets are polarized due to the bromide-rich surface termination. This, in turn, can increase the negative electrostatic potential of the Cs^+^ vacancies on these facets, thus leading to a higher binding affinity of the positively charged dicationic ligands (Figure S2). However, this effect may be less pronounced in real QDs due to surface ion loss or rearrangement. Additionally, variations in facet areas and ligand-binding configurations are not considered in our model (see Supporting Information Note 1 for details). Compared to monodentate primary ammonium ligands, the dicationic ligands generally exhibit adsorption energies that are nearly twice as high (Supporting Information Note 1),^7,31^ which is expected due to their bidentate nature. Note that the calculated adsorption energy of primary ammonium cations might not reflect their actual binding affinity in QD colloids, since solubilization equilibria are not explicitly included in DFT modeling. Furthermore, when each ammonium binding site of the C4 and C6 ligands is considered independently, the calculated adsorption energies are still larger or comparable to those of the single ammonium ligands (Supporting Information Table S1). Therefore, the bidentate C4 and C6 ligands are expected to further stabilize the QD facets compared to monodentate alkylammonium ligands. Our computational results strongly suggest that geometric matching between facets and ligands plays a key role in determining the surface energy of different facets.

The geometric-matching ligand design was experimentally tested by synthesizing dicationic ligands with dodecyl ligand tails and various linker lengths (C3–C7) using a reported method with modifications (detailed in the Methods/Experimental Section).^38^ We applied thermodynamic equilibrium control during QD growth to select the facets, thereby providing a bromide-rich environment required for efficient dicationic ligand binding.^15^ During synthesis, perovskite QD precursors with or without facet-selective ligands are loaded into the reaction flask (details in the Experimental Section). After the hot injection, QDs form and are then annealed at 130 °C to attain their thermodynamically preferred shapes. When QDs are annealed with dicationic ligands, shapes with lower surface energy are favored. As predicted by DFT, the relative binding of C4 and C6 suggests that C4 will stabilize the (100) facet more than C6, while C6 will stabilize the (110) and (111) facets more than C4. This difference in relative adsorption energy agrees with the experimentally observed ligand controlled QD morphologies. The relation between QD morphologies and ligands is illustrated schematically in Figure 1d.

Scanning Transmission Electron Microscopy (STEM) images of perovskite QDs annealed with C4 and C6 ligands confirm their cubical and polyhedral shapes (Figure 2). It can be observed that C4 perovskite QDs self-assemble following a cubic pattern. In contrast, the C6 perovskite QDs form a hexagonal assembly, as demonstrated by the fast-Fourier transform (FFT) patterns (Figure 2a and 2b insets). Indeed, the size dispersion of both C4 and C6 QDs is approximately 10%, suggesting that QDs are monodisperse. The absorption and photoluminescence (PL) spectra of perovskite QDs annealed with C4 and C6 are plotted in Figure 2c. The well-controlled shape of the perovskite QDs is also evident in their absorption spectra: the peak-to-valley ratios for the 1S peak are 1.2 and 1.9 for C4 perovskite QDs and C6 perovskite QDs, respectively. The more distinct absorption peaks in polyhedral perovskite QDs than in cubic perovskite QDs are attributed to their higher symmetry, which has suppressed the mixing and splitting of higher-order absorption states. This is also consistent with previous reports.^14,42^

**Figure 2:**
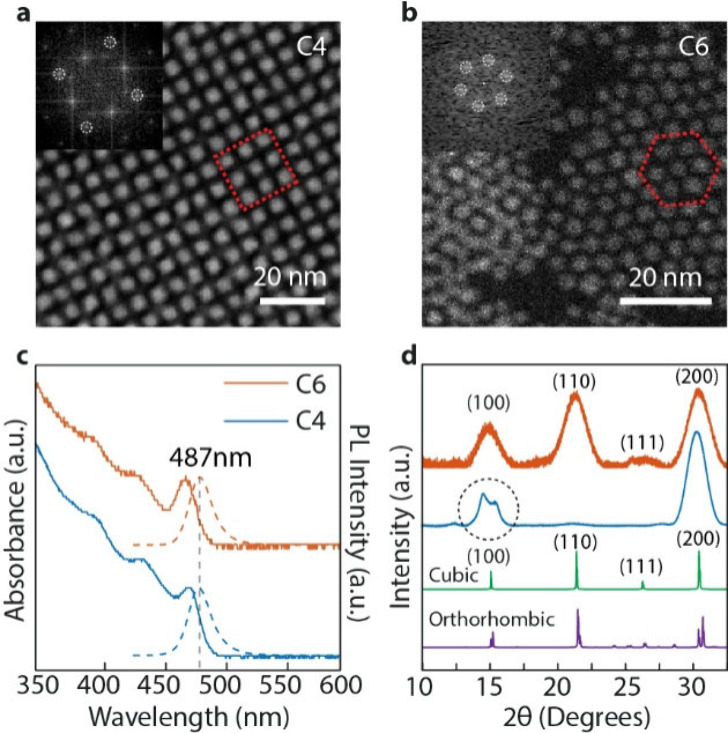
CsPbBr_3_ perovskite QDs with high size and shape uniformity. (a) and (b) STEM images of CsPbBr_3_ QDs annealed with C4 and C6 ligands, respectively. Insets show FFT patterns. (c) Absorption and PL spectra of CsPbBr_3_ QDs annealed with C4 and C6 ligands. (d) XRD patterns of CsPbBr_3_ QDs annealed with C4 and C6 ligands. Also included are the simulated XRD patterns of orthorhombic CsPbBr_3_ (ICSD: 97851) and cubic CsPbBr_3_ (ICSD: 29073) for comparison.

To gain further insights into how well the perovskite QD shape is regulated at an ensemble level, powder X-ray diffraction (XRD) was employed. The XRD patterns of C4 and C6 perovskite QDs are shown in Figure 2d alongside references of the orthorhombic and cubic CsPbBr_3_ XRD patterns. First, the (110) reflection in the C4 perovskite QD sample is nearly completely suppressed, indicating a strong orientation preference along the (100) plane.^43^ This is expected because uniformly cubic perovskite QDs are unlikely to land on their corners or edges on the substrate. Second, the (100) reflection of the C4 perovskite QD splits into at least two fringes (marked in Figure 2d), which result from multilayer diffraction observed to occur in nanocrystal superlattices.^44–46^ It is worth noting that C4 QDs still form superlattices, even though the sample preparation did not involve the slow solvent drying typically required for their formation.^41,46–48^ Such multilayer diffraction has only been observed so far in weakly confined perovskite nanocrystals. Using TEM images of the QDs, the inter-QD spacing was determined to be 2.1 ± 0.4 nm, and the size was determined to be 4.5 ± 0.3 nm, respectively (Figure S3, S4). In comparison, QDs synthesized using the original ligands under the same conditions do not demonstrate multilayer diffraction (XRD in Supporting Information Figure S5). This also suggests that the fringes from (100) reflection are unlikely to stem from the orthorhombic phase of CsPbBr_3_. Overall, C4- and C6-passivated perovskite QDs display high size and shape homogeneity, due to the successful regulation of surface facet exposure. Lastly, the XRD pattern of C6 perovskite QDs shows intense (110) and (111) peaks. The relative intensities of all reflections in the C6 sample are very close to the standard powder XRD pattern, suggesting the C6 QDs are multifaceted and can land on substrates with different facets. Additionally, based on STEM images of C6 QDs, the relative contributions of each exposed facet can be roughly estimated by treating the QD images as projections of polyhedra (Supporting Information Figure S6).

Our experimental results agree well with our DFT predictions: C4 ligands stabilize the (100) facet more than C6, while C6 stabilizes the (110) and (111) facets relative to C4. This supports the experimental results, which show that C4 promotes cubical QD formation, while C6 promotes multifaceted polyhedral QD formation. Since our synthesis method favors thermodynamically stable QDs, annealing the QDs with C4 and C6 ligands is expected to promote the exposure of low-energy facets, thereby influencing surface morphology (Figure 1). To verify the geometric matching effect between ligand and facets, we also synthesized QDs using C3, C5, and C7 ligands and annealing these QDs under the same synthetic conditions. As expected, no clear QD orientation preference nor distinct absorption spectrum features are observed (Supporting Information Figure S7), which supports the proposed ligand-binding-assisted- morphology control mechanism.

Small perovskite QDs are often more susceptible to morphology changes during purification due to weak ligand-surface interaction. In this study, all perovskite QDs were initially washed once with acetone and purified at least once with methyl acetate before characterization, and no significant shape changes were observed (Supporting Information Figures S8, S9). This is attributed to the higher binding affinity of dicationic ligands. ^1^H NMR spectroscopy was used to confirm the ligand-QD binding. Figure 3 compares the ^1^H NMR spectrum of pure C4/C6 ligands with the C4/C6 perovskite QD colloids in toluene-*d*_8_. The α methylene protons in the dodecyl chains of the C4 ligand are observed at 3.52 ppm, and the protons of the *N*-methyl groups are observed at 3.54 ppm (Figure 3a and 3c). These peaks shift in the C4 QD colloids and merge into a notably broadened peak, as shown in Figure 3a, indicating ligand binding. Additionally, the α methylene protons on the linker chain (4.19 ppm) experience an upfield shift to the broadened spectral region circled in Figure 3a, which is consistent with the DFT predicted model since the linker chain is closer to the QD surface (∼2.96–6.24 Å, Supporting Information Figure S10). Similarly, the α methylene protons (multiplet centered at 3.61 ppm) and *N*-methyl protons (3.77 ppm) in C6 ligands are also found in perovskite QD colloids with peak broadening, demonstrating the C6 ligand binding (Figure 3c). It is worth noting that the original oleylammonium ligands also exist on the perovskite QD surface (Figure 3a and 3c). Since both C4/C6 and OAm^+^ can bind to the surface of the QD in close proximity to one another, this may lead to additional peak broadening of the α methylene protons, as the mutual arrangement in adjacent positions can create steric hindrance to rotation.

**Figure 3:**
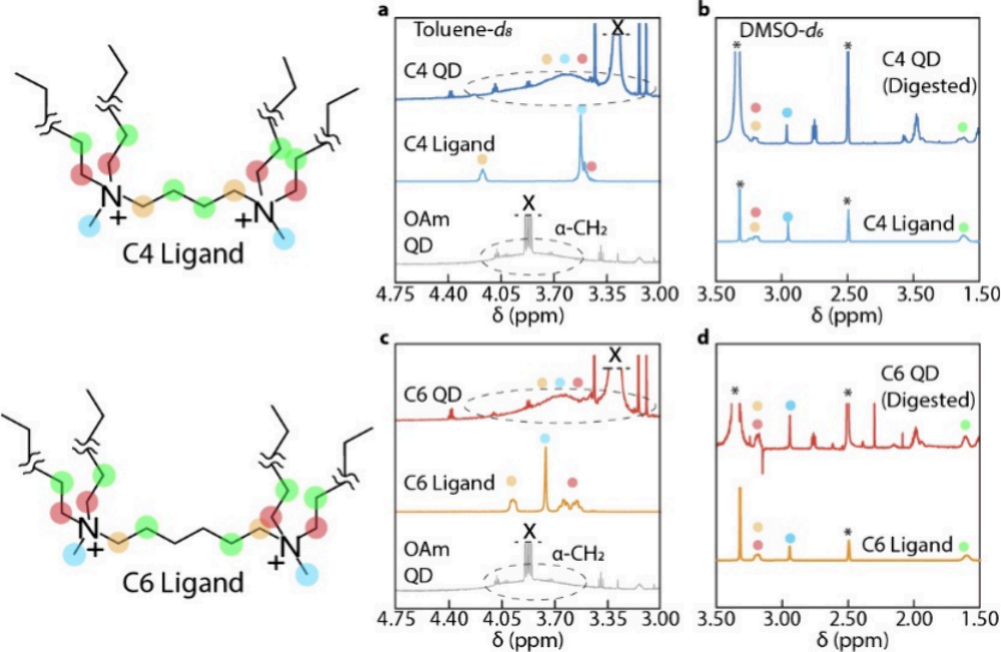
Characterization of the binding of C4 and C6 ligands to the perovskite QDs. (a) and (c) The expanded portions of the ^1^H NMR spectra of pure C4 and C6 ligands and QD samples in toluene-*d*_8_. The OAm-capped QD sample is also included for comparison. (b) and (d) ^1^H NMR spectra of C4 and C6 QD samples digested in DMSO-*d*_6_. The spectra of C4 and C6 ligands alone are shown for comparison. The protons of the methylene groups (CH_2_) in C4 and C6 ligands assigned in the spectra are color-coded in the molecular structure schemes (left). The asterisk indicates proton signals from DMSO at 2.5 ppm and water at ∼3.31–3.55 ppm. The quartet peak, labeled “X”, belongs to the protons of the methylene group of the remaining antisolvent, ethyl acetate, after purification. The peak marked with X at (3.30 ppm) corresponds to the methoxy group of the remaining antisolvent, methyl acetate, after purification. The full (0–8 ppm) ^1^H NMR spectra are given in Supporting Information Figure S12, and the vinyl peak assignment is shown in Figure S13.

To further confirm the binding of the dicationic ligand and to analyze the organic ligands contained in the colloid, perovskite QDs were digested with deuterated dimethyl sulfoxide (DMSO-*d*_6_) (details in the methods section). Figure 3b and 3d compare the ^1^H NMR spectra of pure C4/C6 ligands (in DMSO-*d*_6_) with the digested perovskite QD samples. It is evident that α and β protons, as well as methyl protons from both dicationic ligands, exist in the digested QDs, suggesting that ligands are preserved during purification. Additionally, despite a large amount of zinc bromide being used in the synthesis, elemental analysis (ICP-MS) indicates that the possible remaining Zn^2+^ concentration in the purified QD colloids is very low (Zn/Pb less than 0.05). Note that this includes all possible existence of Zn^2+^ (free ions, lattice dopants, and surface adsorbates). Given the relatively low Zn^2+^ concentration and the confirmed surface dicationic ligand binding (more analyses below), the interference of Zn^2+^ on ligand binding should be insignificant. This is further supported by the observation of shape regulation of C4 QDs using HBr instead of ZnBr_2_ (Supporting Information Figure S11).

After confirming the surface binding of dicationic ligands, we aimed to quantify the ligands in the digested QDs using mesitylene as an internal standard (details in Methods). The concentration of the perovskite QDs was estimated using the previously reported size-extinction coefficient relationship.^49^ For C4 QDs, we identified a molar C4 ligand composition of approximately 13% that can increase to up to 30% after additional antisolvent purification (C4:Oleyl ligands = 0.13–0.3). Note that only about 9% of dicationic ligands (molar ratio) were loaded during synthesis with respect to oleylamine/oleic acid. It is worth noting that while both oleylammonium and oleate ligands are present, the contribution of oleic acid/oleate is relatively small (see Table S3). The higher concentration and good retention of C4 ligands on the QD surface align with their higher binding affinities compared to monodentate oleyl ligands. For C6 QDs, the molar ligand composition of C6 is close to the initial loading ratio of ∼10%. This likely results from the lower binding affinity of C6 ligands on the (100) facets remaining on multifaceted C6 QDs. Considering that ∼67% of oleylammonium cations are bound^12^ (^1^H DOSY NMR results are provided in Supporting Information Figure S14), the actual ligand coverage (dicationic plus oleylammonium cations) of C4 and C6 QDs was determined to be approximately 23% and 55%, respectively. The relatively lower coverage of the C4 QDs correlates with a slightly lower average PLQY (50–70%) compared to the C6 QDs (80 ± 5%) (Figure S15). The decay lifetime of excitons in both the C4 and C6 QDs is comparable to their radiative lifetime (Figure S16),^7^ consistent with the relatively high PLQY. Furthermore, both C4 and C6 QDs display no noticeable shift in their PL peak position under 2 h of continuous laser excitation (Figure S17 and Figure S18). Additionally, postsynthetic addition of ligands can further improve the PLQY of C6 QDs (details in Supporting Information Note 2).

To better understand the mechanism of QD morphology regulation, we tracked the shape changes of perovskite QD ensembles during annealing with C4 and C6 ligands by taking aliquots of the perovskite QD solution for XRD and STEM analysis. These aliquots were individually quenched in an ice bath and reprecipitated as described in the methods section. Figure 4a shows the XRD patterns of C4 perovskite QDs after different annealing times. The relative intensity of the (110) reflection decreases with increasing annealing time, indicating a shift in facet preference from more (110)/(111) to mainly (100). Additionally, fringes from the (100) reflection peak are consistently observed during annealing, suggesting that the high ensemble uniformity remains intact. Similarly, C6 perovskite QDs keep their (110) and (111) exposures during annealing, as indicated by the high intensities of these reflections in the XRD patterns (Figure 4). In contrast, QDs synthesized with the oleyl ligands only show a random orientation, resulting in an XRD pattern exhibiting high intensity (110) and (111) reflections, like that of C6 QDs.

**Figure 4:**
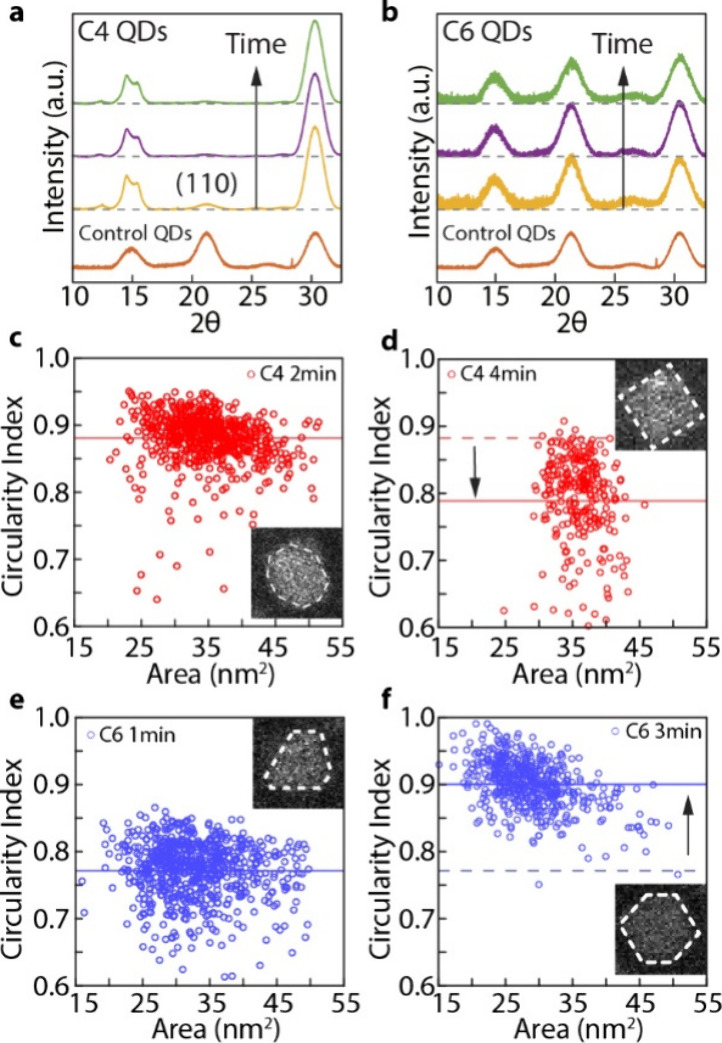
XRD patterns of aliquots of (a) C4-QDs and (b) C6-QDs taken at different annealing times (specified in panels). (c) and (d) Statistics of circularity indices of 360 and 238 C4-QDs after (c) 2 min and (d) 4 min of annealing, respectively. (e) and (f) Statistics of the circularity indices of 341 and 364 C6-QDs at (e) 1 min and (f) 3 min of annealing, respectively. One typical QD STEM image with its morphology outlined is shown for each sample aliquot.

We then examined how dicationic ligands influence the facet exposure of QDs under various synthesis conditions. We chose C4 for these studies because the relative intensities of the (100) and (110) peaks, as well as the multilayer diffraction pattern observed on the (100) peaks in XRD, are highly sensitive to the degree of (100) facet exposure. Although including C4 ligands in the reaction precursors prior to hot injection can facilitate efficient shape control, their initial incorporation is not essential. Figure S19 shows the XRD patterns of QDs at different annealing times with C4 ligands introduced after hot injection. QDs begin to exhibit multilayer diffraction after 6 min, along with a decrease in the (110) peak intensity compared to as-grown QDs occurring at 4 min, confirming the role of dicationic ligands in altering facet exposure after synthesis through annealing. Additionally, the amount of dicationic ligands is crucial for controlling the shape of QDs. In general, a ligand/QD molar ratio of >120 is necessary to promote the exposure of (100) facets (Figure S20). This is expected because increasing ligand coverage widens the free energy difference between QDs by exposing different surface facets, and C4 still needs to compete with the original ligands, despite having a higher binding affinity.

XRD measurements can only reflect the QD orientation preferences at an ensemble level. To gain further insights into the evolution of QD morphology, we employed HAADF-STEM images of aliquots collected during perovskite QD annealing to examine changes in their circularity indices (CIs).^50^ Specifically, statistics on QD sizes and circularity indices for over 200 perovskite QDs in each aliquot have been extracted from the STEM images and summarized in Figure 4. QDs that are free of facet-selective ligands generally show an oblate cuboid shape with a circularity index of ∼0.85 (±0.04). For the synthesis with C4 ligands, the average circularity index increases slightly to 0.86 (±0.05). A closer look at STEM images reveals that the QDs appear as truncated cubes (Figure S21). After approximately 4 min of annealing, the CI of perovskite QDs decreases to 0.77 (±0.08), approaching that of an ideal cube (CI = 0.785). Notably, the average QD area in the STEM images increases from 31 (±5) nm^2^ to 38 (±8) nm^2^, indicating a slight increase in volume during annealing. The PL redshift of perovskite QDs supports this volume increase, as it suppresses quantum confinement effects. This growth can be attributed to the material expanding on the corners of perovskite QDs to maximize exposure of the (100) facets. Meanwhile, the PL peak line width also narrows after annealing due to improved shape uniformity (Figure S22).

Compared with C4 QDs, the CI of C6 QDs after 1 min of annealing is smaller (0.80 ± 0.04). This seemingly surprising result may be interpreted as a change in QD landing facets when they were deposited onto the TEM grid: upon annealing with C6 ligands, more (110) and (111) facets are exposed, and QDs landed on these facets exhibit an irregular hexagon-like shape with a smaller CI (insets in Figure 4). This is supported by the polyhedral QDs observed in the STEM images. After longer-time annealing, the CI increases to 0.89 ± 0.04, indicating improved morphology homogeneity and reduced exposure of (100) facets, as the QD surfaces reach a thermodynamically stable state that maximizes the exposure of (110) and (111) facets.

Our thermodynamic shape regulation can be achieved in very small cubical perovskite QDs (<5 nm), which is usually considered challenging due to the increased surface energy and the purification-induced degradation. Figure S23 shows the XRD pattern of C4 perovskite QDs of ∼4.8 nm, in which the (110) reflection is strongly suppressed, accompanied by a (100) reflection exhibiting three fringes. Fitting the multilayer diffraction fringes suggests an average perovskite QD size of 5.1 nm, which agrees well with STEM measurements (Figures S23, S24).

Size confinement enhances exciton-surface interactions in QDs. We used single-particle spectroscopy to examine exciton properties in cubical and polyhedral QDs. Surface stoichiometry can significantly affect the photostability of quantum-confined QDs. The vacancy formation energy depends heavily on the exposed facets. It has been suggested that point defects form more easily on (110) and (111) facets,^51,52^ which may reduce the photostability of single QDs. Recently, we stabilized single CsPbBr_3_ QDs by epitaxially passivating their (100) facets with molecular crystal matrices.^7^ Using this method, we stabilized C6-single QDs for spectroscopy measurements. However, it is worth noting that some C6 QDs still exhibit spectral diffusion at 3.2 K, and we selected those QDs with relatively stable PL spectra for micro-PL measurements.

We measured single-QD PL spectra at 3.2 K to resolve the fine structure splitting (FSS) of excitons (additional PL spectra of single QDs are provided in Figure S25). Cubical CsPbX_3_ (X = Cl, Br, I) QDs are known to have a triplet bright exciton state, whose degeneracy can be lifted by reduced lattice symmetry, anisotropy, shape-related dielectric discontinuity, and electron–hole exchange interaction or QD size.^53–55^ To avoid interference from the dark exciton and photodegradation, we used slightly larger (6–7 nm) QDs for micro-PL studies. Figure 5 shows the PL spectrum of cubical and polyhedral QDs. While both QDs exhibit a doublet PL spectrum with similar peak energy and width, the FSS of the cubical QD (1.3 meV) is significantly larger than that of the C6 QD (0.42 meV). The C6 QD FSS is also considerably smaller than values reported in the literature for QDs exhibiting doublet PL peaks.^56–58^ Although it is not practical to directly observe the shape of each measured QD, based on its more frequent spectral jumps and the good shape uniformity in our samples, the QD is likely polyhedral. While the reduced FSS in C6 QDs requires further investigation, it may originate from changes in the dielectric environment caused by exposed polar facets or reduced anisotropy compared to cubical QDs. Additionally, previous theoretical studies suggest that a higher degree of anisotropy leads to increased FSS.^59^ In CsPbBr_3_ QDs with an *O*_*h*_ crystal lattice symmetry, the FSS value can rise from approximately 0.6 meV (at 10% shape anisotropy) to about 1.2 meV with 20% shape anisotropy^54^). Overall, the lower FSS values observed in our polyhedral QDs are consistent with these predictions.

**Figure 5:**
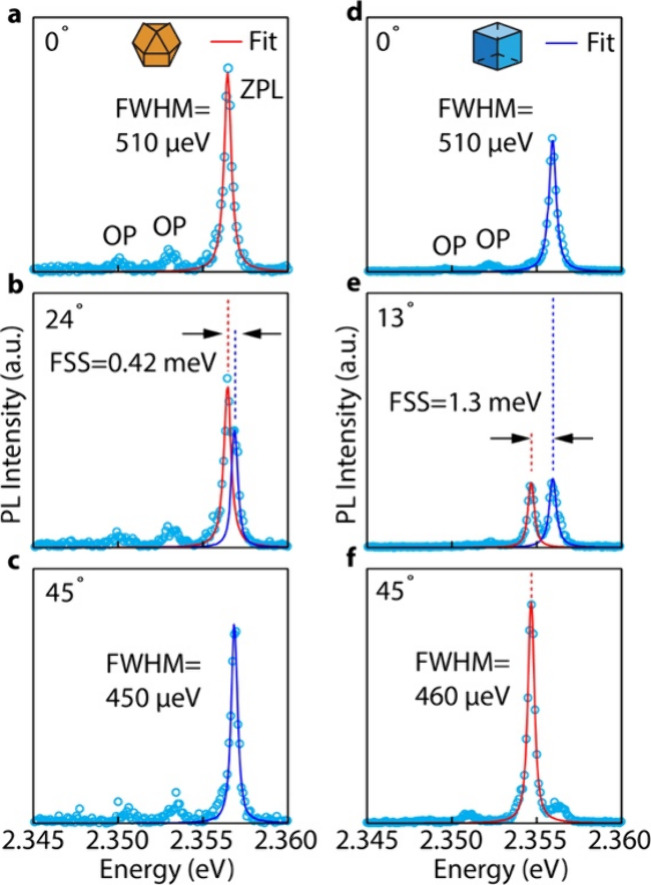
PL spectra of (a–c) polyhedral and (d–f) cubical single QDs. The half-wave plate angle (with respect to the polarizer) is marked in each panel. Both QDs exhibit doublet PL lines with orthogonal polarizations. The phonon side bands are marked in (a) and (d).

In conclusion, a method has been developed to produce perovskite QDs with high uniformity in size and shape. We used dicationic ligands that selectively bind to specific facets based on the spatial matching between the binding groups and vacancy sites. This reduces the surface energy of QDs and exposes those facets. By leveraging different surface energies, we can control their morphology through annealing with dicationic ligands. The uniform shape of perovskite QDs enables rapid self-assembly and clear exciton transitions at the ensemble level. The change in surface facets of the perovskite QDs is mainly driven by thermodynamics, despite fast growth kinetics, offering a route for designing precise perovskite QD syntheses based on the classic Wulff facet principle. Finally, QDs with different surface facet exposure exhibit distinct exciton fine-structure splitting energy, suggesting that the optical properties can be tuned by controlling QD shape.

## Supplementary Material



## Notes

Details on materials and experimental methods, figures showing relaxed configuration of mismatched ligands on different facets, electrostatic potential map of the (100) and (111) facets, inter-QD spacing of C4 and size distribution, XRD pattern and circularity index of CsPbBr_3_ synthesized with no dicationic ligand, 3D models of single C6 QDs, XRD patterns of (C3, C5, C7) QDs, absorption and PL spectra of (C3, C5, C7) QDs, the full spectral range of ^1^H NMR of spectra in Figure 3, PL spectra of C4 and C6 samples for relative PLQY measurements, time-resolved photoluminescence of C4 and C6 samples, ensemble photostability of C4 and C6 QDs, relative PLQY of C4 and C6 QDs under continuous excitation, XRD patterns of C4 QDs with ligand added after Cs-oleate injection, XRD of QDs with insufficient C4 added, STEM images of aliquots of C4 and C6 QDs extracted during annealing, ensemble PL line widths of C4 QDs at (2, 3, and 4 min), multilayer diffraction fitting of QDs, μ-PL spectra of additional spheroidal QDs at 3.2 K; tables containing adsorption energies of ligands in different configurations, relative PLQY of sample after ligand exchange, estimated ligand concentrations of QDs (PDF)
