# Supplementary material for: Controlling the Surface Morphology of Strongly Confined CsPbBr3 Perovskite Quantum Dots
Source: Nano Lett. 2026 Jul 15;26(29):9418–26. doi: 10.1021/acs.nanolett.6c01367 (PMC13430681; doi:10.1021/acs.nanolett.6c01367)
Supplement: Supplementary file 1 [file nl6c01367_si_001.pdf]

Supporting Information

Controlling the Surface Morphology of Strongly Confined $CsPbBr_3$ Perovskite Quantum Dots

Matthew L. Atteberry[1], Chance Lander[1], Carly Wickizer[1], Amila Wanasinghe[1], Chenjia Mi[1], Novruz G. Akhmedov[1], Sisi Xiang[2], Gavin C. Gee[3], Heath Hadley[4], Parker H. Bryan[1], John W. Peters[1], Madalina Furis[4,5,6], Yihan Shao[1], Yitong Dong[1,5,6*]

[1]Department of Chemistry and Biochemistry, University of Oklahoma, Norman, Oklahoma 73019, USA

[2]Materials Characterization Facility, Texas A&M University, College Station, Texas 77843, USA

[3]School of Materials Science and Engineering, The University of Oklahoma, Norman, OK 73019, USA

[4]Homer L. Dodge Department of Physics and Astronomy, University of Oklahoma, Norman, Oklahoma 73019, USA

[5]Center for Quantum Research and Technology, The University of Oklahoma, Norman, Oklahoma 73019, USA

[6]Materials Science and Engineering Program, The University of Oklahoma, Norman, Oklahoma 73019, USA

Corresponding to:

Yitong Dong, Email: Yitong.Dong-1@ou.edu

Table of Contents

Experimental Section S4-S9

Materials S4

Cs-Oleate Synthesis S4

Morphology Controlled $CsPbBr_3$ QD synthesis S4-5

Dication free QD synthesis S5

$ZnBr_2$ free QD synthesis S5-6

Dicationic ligand synthesis S6

Structural Characterization S6

ICP-MS S6

Optical Characterization S7

Single-Particle Measurements S7

Nuclear Magnetic Resonance Measurements S7-8

1H DOSY Bound Fraction Calculations S8-9

Computational Methods S9

Supporting Information Note 1 .... S10

Table S1 .... S10

Figure S1 .... S10

Table S2 .... S11

Figure S2 .... S11

Figure S3 .... S11

Figure S4 .... S12

Figure S5 .... S12

Figure S6 .... S12

Figure S7 .... S13

Figure S8 .... S13

Figure S9 .... S13

Figure S10 .... S13

Figure S11 .... S14

Figure S12 .... S14

Table S3 .... S14

Figure S13 ... **S15**
Figure S14 ... **S15**
Figure S15 ... **S15**
Figure S16 ... **S15**
Figure S17 ... **S16**
Figure S18 ... **S16**
Supporting Information Note 2 ... **S16-17**
Table S4 ... **S17**
Figure S19 ... **S17**
Figure S20 ... **S18**
Figure S21 ... **S18**
Figure S22 ... **S18**
Figure S23 ... **S19**
Figure S24 ... **S19**
Figure S25 ... **S19**
Supporting Information Note 3 ... **S19-21**
Table S5 ... **S21**
References ... **S22-23**

Experimental Section

Materials

Cesium carbonate (Puratronic 99.994%, Alfa Aesar), lead (II) bromide (Puratronic, 99.999% Alfa Aesar), zinc bromide (metals basis 99.9%, Alfa Aesar), oleylamine (OAm, technical grade, 70%, Sigma-Aldrich), oleic acid (OA, technical grade, 90%, Sigma-Aldrich), 1-octadecene (ODE, technical grade, 90%, Sigma-Aldrich), acetone (certified ACS, Fisher), hexanes (HPLC grade, Millipore), methyl acetate (ReagentPlus, 99%, Sigma-Aldrich), chloroform (ACS reagent grade, Macron), acetonitrile (99.9%, Sigma), diethyl ether (≥99.7%, Anhydrous, Sigma-Aldrich), N,N-didodecylmethylamine (>85.0%, TCI), 1,3-dibromopropane (ReagentPlus, 99%, Sigma-Aldrich), 1,4-dibromobutane (99%, Sigma-Aldrich), 1,5-dibromopentane (97%, Sigma-Aldrich), 1,6-dibromohexane (96%, Sigma-Aldrich), 1,7-dibromoheptane(98%, Ambeed), Chloroform-d (99.8%, Cambridge Isotopes), DMSO-d_6 (99.8%, Cambridge Isotopes), didodecyldimethylammonium bromide (DDAB, >98%, TCI), Coumarin 102 (98%, Sigma-Aldrich), Ethyl Alcohol Anhydrous (Pharmco, 100%), Hydrobromic Acid (48 wt% in water, Acros Organics), Toluene-d_8 (99.5%, Cambridge Isotopes), Ethyl Acetate (99.8%, Acros Organics) were used as received.

Cs-Oleate Synthesis

To a 50 mL 3-neck flask, 600 mg of Cs_2CO_3 and subsequently pumped under vacuum and purged with nitrogen 3 times. Upon completion, the flask was placed under nitrogen and subsequently 2.4 mL of oleic acid (OA) and 6.4 mL of octadecene (ODE) were added and subsequently placed under vacuum. The flask was degassed under vacuum for 20-30 minutes at room temperature then subsequently heated under vacuum to 120 °C until the reaction was completed. The flask was then placed under an inert nitrogen atmosphere and kept at a constant 100 °C for further use. The total amount of Cs-oleate precursor can be scaled up or down linearly as needed.

Morphology Controlled $CsPbBr_3$ QD synthesis

The synthesis of the quantum dots with diammonium was based on a modified version of a previously reported synthesis.[1] In short, 300 mg of $PbBr_2$, 700 mg of $ZnBr_2$ (or 600 mg of $PbBr_2$ and 1400 mg of $ZnBr_2$ in the case of larger scale reactions) were added to a 100 mL 3 necked flask. Identical to the Cs-oleate precursor, 3 pump-purge cycles were preformed prior to the addition 6 mL of OA, 6 mL of OAm, and 20 mL of ODE were added to the flask (double these for larger scale reactions). The mixture was degassed at room temperature for 5 minutes under vacuum then subsequently heated to 150°C under vacuum until all solids have dissolved. Upon dissolution, the flask was placed under nitrogen and cooled to 130°C. Subsequently, a septum was removed from the flask and the N_2 pressure was raised to maintain a positive pressure. Then, 0.25-3.7g of the selected dicationic ligand was added to the flask. Typical reaction conditions used 1.5g of the chosen dicationic ligand. Upon addition, the septa was reattached and the flask

was briefly placed under vacuum, then refilled with N_2. To trigger the reaction 2-2.4 mL (4-4.5 mL for larger scale reactions) of Cs-Oleate was injected into the lead precursor containing flask. The particles were annealed at 130 °C for at least 3 minutes then the reaction was subsequently quenched with an ice bath. For reactions where aliquots were taken, scaled-up reactions were performed and 10 mL of the crude QD solution was extracted utilizing a syringe and quenched individually in an ice bath. Larger C6 QDs were synthesized by raising the reaction temperature to 180 °C and reducing the annealing time reduced to 1 minute. The QDs were subsequently loaded into centrifuge tubes and centrifuged at 7300 RPM for 10 minutes, and the supernatant collected. To the supernatant a 1:1 volume acetone saturated with NaBr was added and centrifuged at 7300 RPM for 5 minutes and the precipitant collected, and the supernatant discarded. The QDs were resuspended in 3-5 mL of hexane or chloroform and a 3:1 volume ratio of methyl acetate was added to the solution. These were subsequently centrifuged at 7300 RPM and the precipitate collected and resuspended in 1-2 mL of hexane for further characterization. For larger QDs the precipitants were collected after initial centrifugation and suspended in hexane and centrifuged at 7300 RPM for 5 minutes and the supernatant collected.

Dicationic free QD synthesis

Dicationic free $CsPbBr_3$ QDs were synthesized using the method reported by Protesescu *et al.*[2] with several modifications. In short, 200 mg of Cs_2CO_3 was loaded into a 3 necked 50 mL flask and evacuated and refilled with nitrogen 3 times. Upon completion 7.5 mL of ODE and 0.6 mL of OA were added, and 3 more pump-purge cycles were performed. The flask was placed under vacuum for 15 minutes, then heated to 120 °C under vacuum until a clear, colorless solution was formed and no bubbling was observed. This was placed under nitrogen and stored at 120 °C until use. To a 100mL 3 necked flask 110 mg of $PbBr_2$ was added and 3 pump-purge cycles were performed. To this, 10 mL of ODE, 1.2 mL of OA, and 1.3 mL of OAM were added and 3 more pump-purge cycles performed. Under nitrogen the flask was heated to 195 °C, upon which 0.9 mL of the Cs precursor was injected to initiate the reaction. The reaction was cooled to 180 °C using compressed air then quenched in an ice bath. The crude solution was centrifuged for 5 minutes at 7830 RPM and the precipitants resuspended in 1-2 mL of hexane. This solution was centrifuged at 15000 RPM for 3 minutes the supernatant collected and precipitants discarded.

$ZnBr_2$ free QD synthesis

The synthesis of the quantum dots with diammonium utilized 300 mg of $PbBr_2$ was loaded into a 3 necked flask and 3 pump-purge cycles were preformed prior to the addition 4 mL of OA, 4 mL of OAm, and 20 mL of ODE were added to the flask. To this, 200 µL of HBr was added to the mixture and allowed to stir for 5 minutes under nitrogen. The mixture was degassed at room temperature for 5 minutes under vacuum then subsequently heated to 150°C under vacuum until all solids have dissolved. Upon dissolution, the flask was placed under nitrogen and cooled to 140°C. Subsequently, a septum is removed from the flask and the N_2 pressure was raised to maintain a positive pressure. Then, 0.4g of C4 was added to the flask. To trigger the reaction 1.8

mL of Cs-Oleate was injected into the lead precursor containing flask. The particles were annealed at 130 °C for at least 1 minute then an additional 0.4g of C4 was added. After 3 more minutes the reaction was subsequently quenched with an ice bath. The QDs were subsequently loaded into centrifuge tubes and centrifuged at 7300 RPM for 10 minutes, and the supernatant collected. To the supernatant a 1:1 volume acetone was added and centrifuged at 7300 RPM for 5 minutes and the precipitant collected, and the supernatant discarded. The QDs were resuspended in 3-5 mL of hexane and a 3:1 volume ratio of methyl acetate was added to the solution. These were subsequently centrifuged at 7300 RPM and the precipitate collected and resuspended in 1-2 mL of hexane for further characterization.

Dicationic Ligand Synthesis

The procedure for the synthesis of the dicationic ligands is adapted from a procedure previously reported by the Bawendi group.[3] To a 100 mL 1-neck round bottom flask 40 mmol of N,N-didodecylmethylamine, 10 mmol of the chosen dibromoalkane and 25 mL of acetonitrile were added. This flask was attached to a reflux condenser and placed in a heating block and allowed to reflux for 20 hours under a nitrogen atmosphere. Purification deviated from the initially reported procedure; upon completion of the reaction, the flask was cooled in an ice bath and diethyl ether was added to the crude solution upon which a white precipitate was observed to crash out. The precipitate was collected via vacuum filtration and washed >5 times with diethyl ether. The purified white solid was collected and dried overnight in a vacuum oven before further use.

Structural Characterization

Scanning Transmission electron microscopy images of all QDs were obtained using a Titan Themis 300 TEM microscope operated at 300 kV. Image analysis was performed with ImageJ, with the Fiji distribution.[4] Segmentation of the STEM images was conducted using Trainable Weka Segmentation (v4.0.0).[5] The area and circularity of the $CsPbBr_3$ QDs were subsequently measured using the "Analyze Particles" function with restrictions on the size and circularity ranges to avoid measuring aggregated particles. The equation (eq. S1) for circularity is defined as follows:

$$Circularity\ Index = 4\pi \frac{Area}{Perimeter^2} \qquad (S1)$$

XRD patterns for the $CsPbBr_3$ QDs were collected on a Rigaku SmartLab X-ray diffractometer with a Cu-Kα source. Samples were prepared by drop casting the stock QD colloids directly onto a clean silicon substrate ~10×10 mm in size.

ICP-MS

The composition of the C4 and C6 QDs was determined using an Agilent 7850 ICP-MS. The QD solutions were digested in concentrated nitric acid for ICP-MS analyses.

Optical Characterization

Linear absorption and photoluminescence (PL) spectra were collected using an Ocean Insight Maya 2000 spectrometer. A 385 nm LED was used as the excitation source for PL measurements. The PQD stock solutions were typically diluted 100–1000 times in hexanes or chloroform for the measurement. The PLQY of the C4 and C6 QDs was measured with respect to a Coumarin 102 dye sample (76.4% Quantum Yield), both excited with an OBIS LX SF 405 nm continuous-wave laser (Coherent). QDs were suspended in hexane and diluted to an optical density of 0.07 O.D. respectively, and the difference in solvent refractive indices was accounted for when calculating the PLQY.

Time resolved photoluminescence (TRPL) measurements were performed using a time-correlated single-photon counting technique. The colloidal sample in a cuvette was excited with a 405 nm pulsed laser (Picoquant LDH-D-C-405) driven by a Picoquant Sepia PDL828 module at a 5 MHz repetition rate and detected using a single-photon avalanche photodiode (Hamamatsu C11202-100). The photon arrival time was recorded using a Picoquant HydraHarp 400 correlator.

Photostability measurements were performed by diluting C4 and C6 QDs to 0.1 O.D. in hexane and monitoring the PL over a period of 2 hours under continuous excitation from a OBIS LX SF 405 nm continuous-wave laser at an irradiance of 49.4 mW/cm^2

Single Particle Measurements

The single particle micro PL samples were prepared on silicon substrates following a previously published method with modifications.[6] The cryogenic single particle measurements were carried out at 3.2 K in a cryostat (Montana Instrument CryoAdvance 50) with a custom built epi-illuminating fluorescence microscope system. The QDs were excited with a 405 nm cw laser (Picoquant LDH-D-C-405) focused by an air objective (Zeiss EC EPN 100×, NA 0.9, vacuum compatible). The single-particle PL was collected by the same objective, passed through a filter set to remove reflected laser, sent through a polarization module consisting of a zero-order half-wave plate mounted on a rotation mount and a fixed angle linear polarizer, then measured by a spectrograph (Andor Shamrock 500i, 1200 l/mm grating blazed at 500 nm) consisting of an EMCCD camera (Andor iXon Ultra 897).

NMR (Nuclear Magnetic Resonance) measurements

The ^1H NMR spectra were acquired at a probe temperature of +25˚C on a 500 MHz VNMRS instrument (operating at 499.8321 MHz) equipped with a 5 mm PFG (z-axis pulsed field gradient) indirect detection probe. Typical parameters for acquiring ^1H NMR spectra were as follows: spectral width 7807.16 Hz, acquisition time 4.0 s, pulse width 3.16 μs (45˚), relaxation time 2 s, and number of transients 32.

The raw FIDs were processed without any apodization prior to Fourier transformation. The chemical shifts of the protons in the ^1H chemical spectra are given in parts per million (ppm) and

are referenced to the residual proton peak of the solvents used (7.26 ppm for $CDCl_3$, 2.50 ppm for DMSO-d_6, 2.09 ppm for toluene-d_8). For estimating the concentration of the stock $CsPbBr_3$ were diluted in hexane 100-500 times depending on the stock concentration and the absorption spectrum was recorded. Utilizing previously reported extinction coefficients, the concentration of the $CsPbBr_3$ was estimated.[7] Subsequently, 0.6-1.8 mL of the purified $CsPbBr_3$ colloid was loaded into an NMR tube and the hexane was removed under vacuum. To the dried $CsPbBr_3$ QDs, 700 µL of DMSO-d_6 spiked with 0.4 µl of mesitylene as an internal standard was added, leading to the digestion of the $CsPbBr_3$ QDs and the release of surface bound ligands. The concentration of the diammonium ligands was determined by integrating the characteristic resonances corresponding to the N-methyl group of C4 and C6 diammonium ligands relative to the aromatic protons of mesitylene at 6.76 ppm. The oleylammonium concentration was determined by integrating the resonance of the vinyl protons at 5.32 ppm. Note that the vinyl proton peak can also be contributed to by oleate anions that remain in the colloid, this may result in underestimation of the dicationic ligand ratios. Utilizing the known concentration of the $CsPbBr_3$ stock solutions and the average sizes from STEM imaging we estimate the ligand coverage of our QDs as summarized in (Table S5).

^{1}H DOSY bound fraction calculation

The ^{1}H DOSY experiments were performed utilizing a Bipolar Pulse Pair Stimulated Echo with Longitudinal Eddy Current Delay (BPP_STE_LED) pulse sequence. The gradient pulse duration was held constant while the amplitude (maximum of 300 mT/m) was systematically varied. The diffusion time used was (Δ = 200 ms) and a gradient pulse length (δ = 3 ms). The diffusion coefficient of the oleyl ligands was determined using the Stejskal–Tanner equation (eq. S2), using the well-resolved vinyl proton resonance.

$$I = e^{-D_{measured}\,\gamma^2\,\delta^2\,g^2\,(\Delta-\delta/3)} \qquad \text{(S2)}$$

In this equation, D is the diffusion coefficient, γ is the gyromagnetic ratio, and g is the gradient strength. All other terms are fixed under the utilized experimental conditions, allowing for the diffusion coefficient to be determined.

This diffusion coefficient is a function of both the free ligand diffusion coefficient (D_{Free}) and that of the colloid (D_{Bound}). D_{bound} was calculated using a derivation of the Stokes-Einstein equation (eq. S3)[8]. D_{free} can be obtained by measuring the free ligand at sub-critical micellar concentrations.

Once both the diffusion coefficients of the colloid and the free ligands are determined, the bound fraction (*f*) can be readily determined using eq. S4.[9, 10]

$$D_{bound} = \frac{K_B T}{6\pi\eta 0.66d} \tag{S3}$$

Here, d is the edge length of the QD, η is the solvent viscosity, T is absolute temperature, and K_B is the Boltzmann constant.

$$f = \frac{D_{free} - D_{measured}}{D_{free} - D_{bound}} \tag{S4}$$

Computational Methods

The $CsPbBr_3$ surfaces were constructed using crystal structure data from ICSD 201285.[11] The Atomistic Simulation Environment (ASE)[12] was used to construct 4x4x4 supercells with Cs-Br rich (100), (110), and (111) facets. From the pristine supercell, two surface Cs atoms were removed and replaced with a dicationic ligand resulting in a charge neutral system. A 10 Å vacuum layer was added to both sides of the adsorption axis to separate the tail of the ligand from the perovskite layer in the next layer of the periodic cell. For each ligand-surface system, the adsorption energy was calculated using three sets of calculations: (i) the ligand-surface complex, (ii) the surface with 2Cs and 2Br vacancies, and (iii) the ligand with 2 Br anions.

The adsorption of the C4 and C6 dicationic ligands to the CsPbBr3 facets was modeled using the CP2K 2024.2 software package.[13] Ligand-surface complexes were optimized using the PBE functional[14] with the TZVP MOLOPT-UZH basis set[15, 16] and the GTH-PBE pseudopotential.[17-19] Dispersion interactions were accounted for using Gimme's D3 dispersion model with Becke-Johnson (BJ) damping.[20, 21] The orbital transformation method with the conjugate gradient algorithm and full-kinetic preconditioner with a 2-point line search was used to converge the SCF energy below a threshold of 10^{-6}.[22-25] An energy cutoff of 400 Ry and a relative energy cutoff of 80 Ry with five grids was used for all CP2K calculations. Geometry optimizations were performed using the BFGS algorithm as implemented in ASE until the maximum force on each atom was below 0.01 eV/Å. The coordinates of all perovskite atoms (Cs, Pb, and Br) were fixed during geometry optimization. After optimization of the ligand position, single point energies were calculated using a dipole correction along the adsorption axis and with a counterpoise correction to reduce basis set superposition error.

Supporting Information Note 1:

In our DFT model, the perovskite crystal facets are terminated by Cs and Br ions and are treated as rigid. Figure S1 illustrates three examples of binding configurations between geometrically mismatched ligand-facet pairs. In Figure S1a, the C6 linker must be distorted to span the nearby Cs vacancies on the (100) surface. In contrast, Figure S1b and C shows one C4 linker methyl group on the ammonium cations away from the binding pocket of the (110) and (111) facets, resulting in a lower binding affinity.

Table S1. A list of adsorption energies of different ligands to the (100) facet of $CsPbBr_3$

Ligand	E_ads (eV)	Reference
DDAB	2.37	*Nat. Commun.* **2025,** *16,* 204
PEABr	2.00	*Nat. Commun.* **2025,** *16,* 204
Cs-Oleate	2.22	ACS Energy Lett. **2019,** *4,* 819–824
CsBr	2.27	*J. Am. Chem. Soc.* **2024,** *146,* 20636–20648
RNH_3^+	1.86	*J. Am. Chem. Soc.* **2024,** *146,* 20636–20648
DDMAB	2.09	*ACS Energy Lett.* **2019,** *4,* 819–824
C4	5.26	This Work
C4 per N^+	2.63	This Work
C6	4.74	This Work
C6 Per N^+	2.37	This Work

Supporting Information Figures:

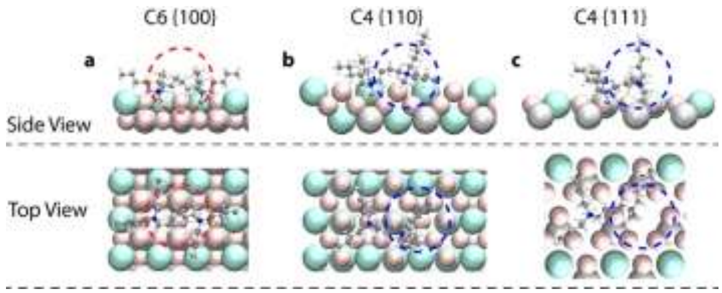

Figure S1. Relaxed configurations of mismatched ligand-facet combinations: (a) C6 ligands attached to (100), (b) C4 ligands attached to (110) facets, (c) C4 ligands attached to the (111) facet. The red circle indicates the distorted linker when it is longer than the Cs-vacancy spacing. The blue circles indicate the dislocated ammonium group when the linker length is shorter than the Cs-vacancy spacing on the (110) and (111) facets. Cyan, grey, and magenta spheres represent Cs^+, Pb^{2+}, and Br^- ions, respectively.

Table S2 Calculated energies for the ligands, surfaces, ligand-surface complexes, and the corresponding adsorption energy for the binding of C4 and C6 to the (100), (110), and (111) facets. Energies are dipole and counterpoise corrected. Cs-Cs distances are provided in Å.

	Calculated Energies				
Complex	Adsorption (eV)	Complex (au)	Surface (au)	Ligand (au)	Cs-Cs Distance (Å)
c4 (100)	-5.257	-4273.939	-4044.556	-229.190	5.874
c4 (110)	-2.400	-4271.683	-4042.405	-229.190	8.307
c4 (111)	-5.401	-4272.094	-4042.706	-229.190	8.307
c6 (100)	-4.731	-4287.691	-4044.556	-242.961	5.874
c6 (110)	-3.043	-4285.478	-4042.405	-242.961	8.307
c6 (111)	-5.923	-4285.884	-4042.706	-242.961	8.307

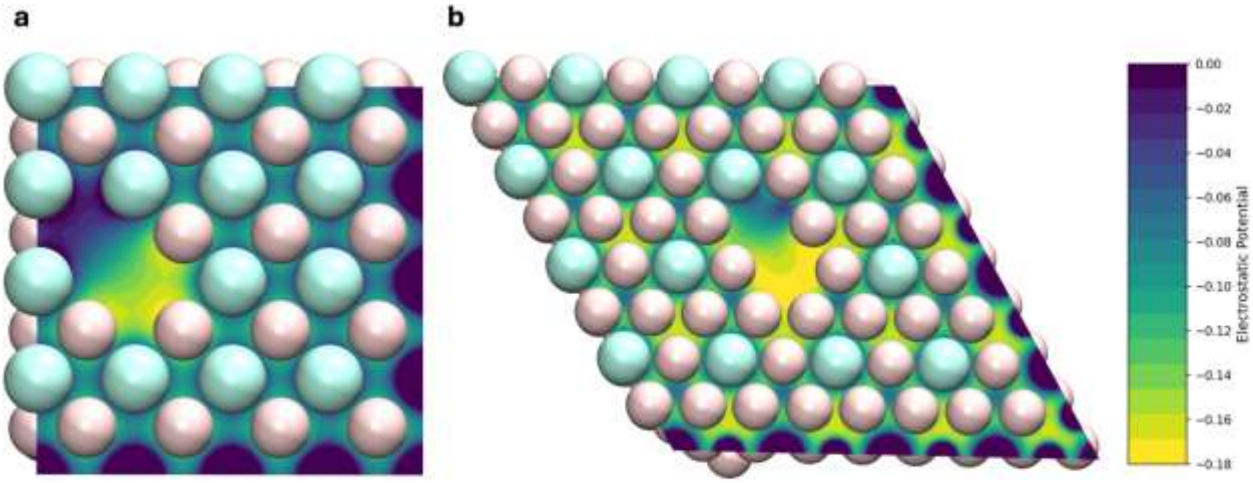

Figure S2. Electrostatic potential (ESP) map of the (a) (100) facet and (b) (111) facet with one Cs^+ and one Br^- vacancy. Cyan and magenta spheres represent Cs^+ and Br^- ions, respectively. Pb atoms are not shown for clarity. The electrostatic potential is visualized using the Volume Slice representation in VMD[26].

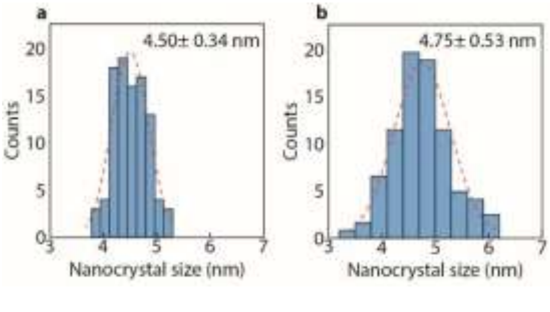

Figure S3. Size distributions of (a) C4 and b (C6) QDs shown in Figure 2.

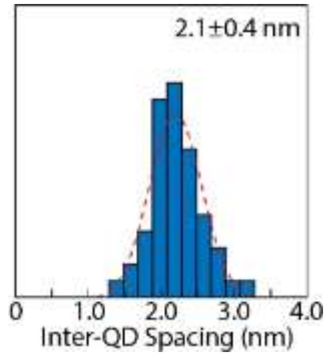

Figure S4. Interparticle spacing between 100 C4 QDs.

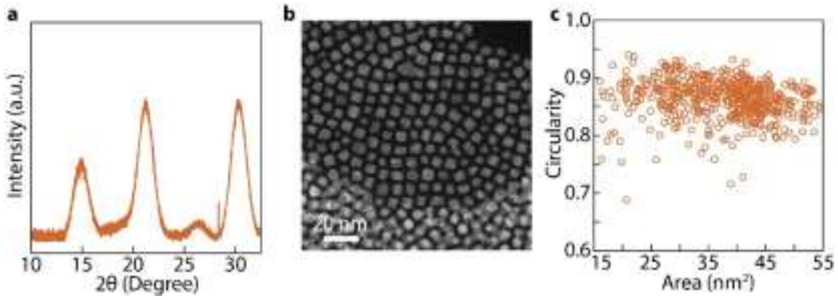

Figure S5. (a) XRD pattern, (b) circularity indices of $CsPbBr_3$ QDs synthesized without adding dicationic ligands.

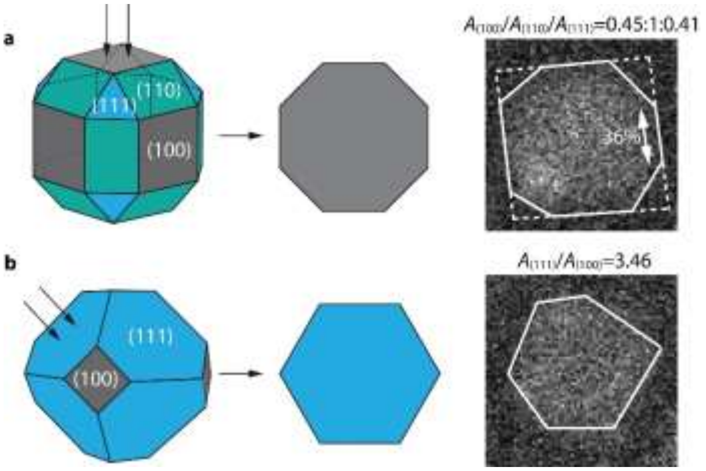

Figure S6. 3D models of C6 QDs exposing (110) and (111) facets, along with their 2D projections following the direction marked by the arrows. On the right, STEM images of two individual C6 QDs are shown, which resemble their corresponding projections. The area ratio of the exposed facets is 2.45 [(110)/(111)] for (a) and 3.46 [(111)/(100)] for (b).

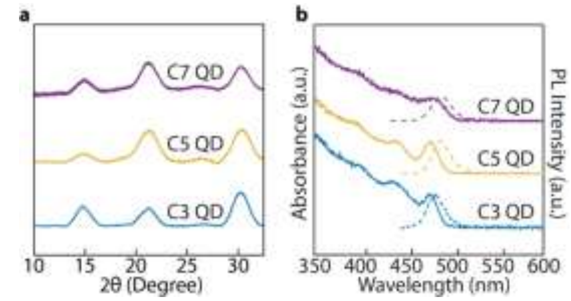

Figure S7. (a) XRD patterns of QDs synthesized with the addition of C3, C5, and C7 ligands and (b) their corresponding PL and absorption spectra.

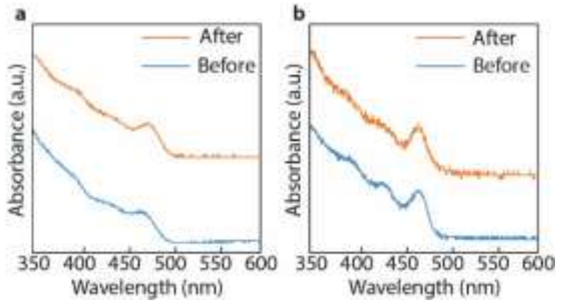

Figure S8. Absorption spectra of (a) C4 QDs and (b) C6 QDs before and after washing.

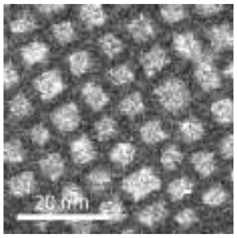

Figure S9. STEM image of C4 QDs without purification using methyl acetate.

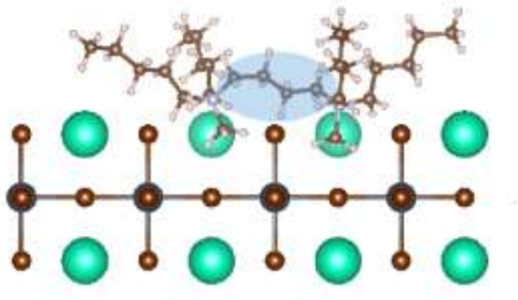

Figure S10. Optimized structure of C4 ligand binding to the (100) facet of $CsPbBr_3$.

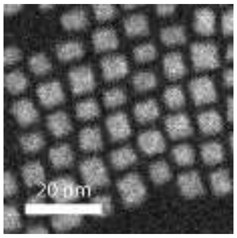

Figure S11. STEM image of $CsPbBr_3$ QDs synthesized with C4 utilizing HBr as an additional Br^- source.

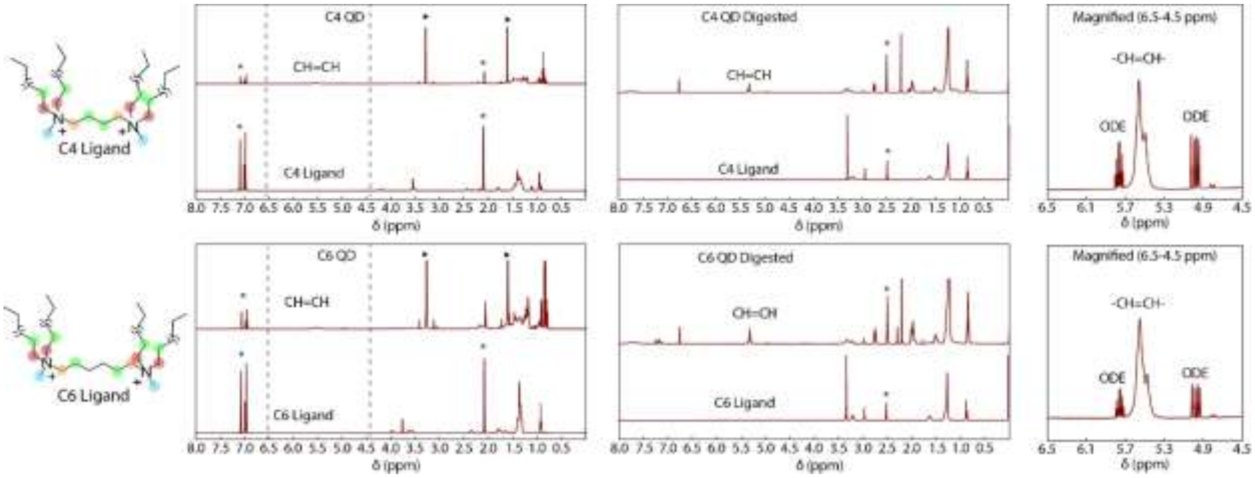

Figure S12. Expanded spectral range of the ^{1}H NMR spectra contained in figure 3. The asterisks denote the residual proton peaks of toluene-d_8 (2.09 ppm and 7.09 ppm) and DMSO-d_6 (2.50 ppm). The triangles mark methyl acetate originating from reprecipitation of the QDs (1.62 ppm and 3.30 ppm). The ^{1}H spectra contain impurity peaks corresponding to octadecene containing a terminal vinyl group (-$CH=CH_2$). The molecular formula of 1-octadecene is $CH_2=CH(CH_2)_{15}CH_3$. The protons of interest in the group are shown in the expanded structure of the vinyl fragment of 1-octadecene in Figure S13. The expanded region of the spectra from 6.5-4.5 ppm of the C4 and C6 QDs in toluene is shown.

Table S3. Estimated ligand concentrations of $CsPbBr_3$ QDs. Note that two QDs samples have different concentrations.

Concentration	C4 Sample	C6 Sample
Mesitylene	3.6×10^{-3} M	3.6×10^{-3} M
Oleylammonium	8.8×10^{-4} M	5.1×10^{-3} M
Oleate	2.4×10^{-4} M	1.2×10^{-5}M

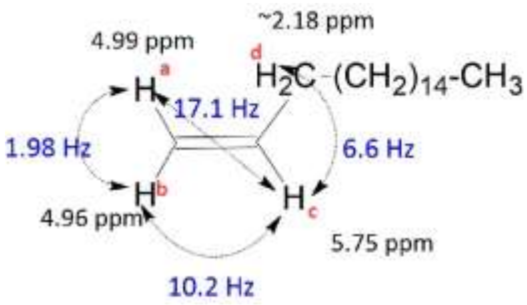

Figure S13. Expanded structure of the terminal vinyl group of 1-octadecene (ODE).

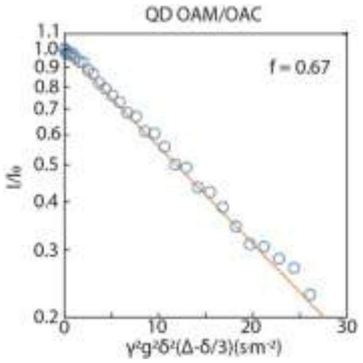

Figure S14. ^{1}H DOSY NMR of $CsPbBr_3$ synthesized with only oleylamine and oleic acid. The bound fraction of oleylamine to the QDs was determined to be f = 0.67.

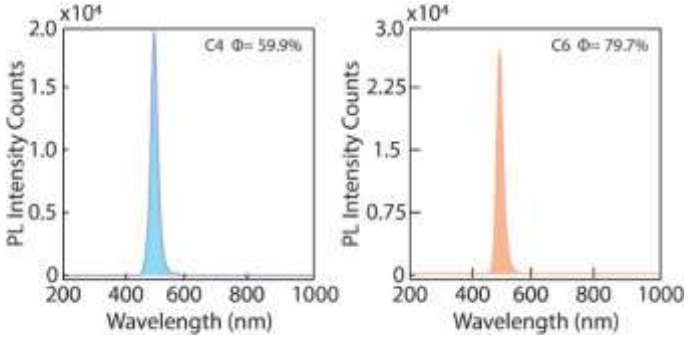

Figure S15. PL spectra of C4 and C6 samples for relative PLQY measurements.

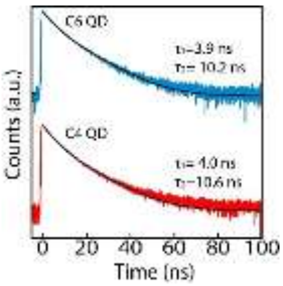

Figure S16. TRPL of QDs synthesized with C4 and C6.

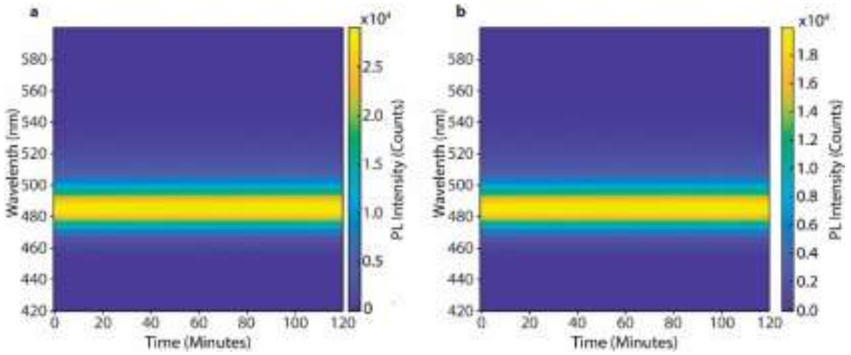

Figure S17. Ensemble photostability of (a) C6 and (b) C4 QD samples under continuous laser excitation for 2 hours.

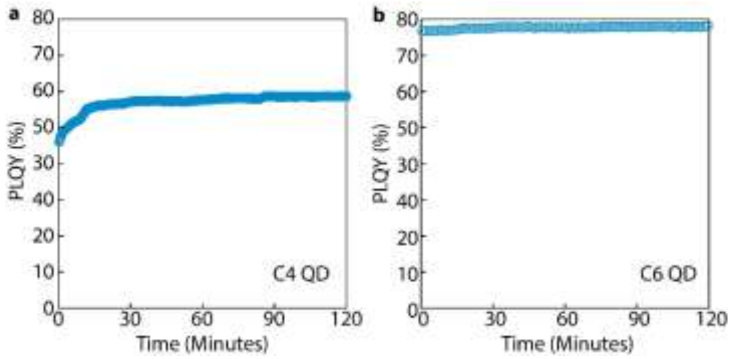

Figure S18. Relative PLQY under continuous photoexcitation for (a) C4 QDs and (b) C6 QDs.

Supporting Information Note 2 Ligand exchange effect on quantum yield.

C4 and C6 QDs were diluted into hexane, and the absorption spectrum were recorded. Utilizing the size dependent optical cross section of $CsPbBr_3$ QDs and having determined the size of the ensembles from STEM imaging, the molar extinction coefficient for the QDs can be determined using the equation (eq. S5)

$$\sigma_\lambda = \varepsilon_\lambda \frac{2303}{N_A} \qquad (S5)$$

where (σ_λ) is the absorption cross section at a specific wavelength, (ε_λ) is the molar extinction coefficient with (N_A) being Avogadro's number. This allows for the concentration of the colloid to be determined via Beer's Law where (eq. S6),

$$A_\lambda = \varepsilon_\lambda b c_{QD} \qquad (S6)$$

Where (A_λ) is the absorbance at a given wavelength, (ε_λ) is the molar extinction coefficient at that wavelength, b is the pathlength, and (c_{QD}) is the concentration of the QD colloids.[7]

Stock ligand solutions containing DDAB, C4, and C6 were made at concentrations of 5 mg/mL by dissolving the DDAB into hexanes and the C4 and C6 into 98% hexanes and 2% anhydrous ethanol by volume**.** To 0.5 mL of the stock QD colloid 100 and 200 equivalents of either DDAB or dicationic ligand solution were added respectively the absorption spectrum recorded and PL spectrum taken the results of this ligand exchange are summarized in table S4.

Table S4. Relative Quantum PLQY of samples post ligand exchange corresponding to samples in figure S18.

Sample	PLQY	ΔPLQY
C4-Stock	46.6%	----
C4-100 equivalents C4	32.8%	-13.8%
C4-100 equivalents DDAB	26.2%	-20.4%
C4-200 equivalents C4	34.7%	-11.9%
C4-200 equivalents DDAB	34.6%	-10.0%
C6 Stock	77.1%	----
C6-100 equivalents C6	89.2%	+12.1%
C6-100 equivalents DDAB	86.6%	+9.5%
C6-200 equivalents C6	68.4%	-8.7%
C6-200 equivalents DDAB	61.8%	-15.3%

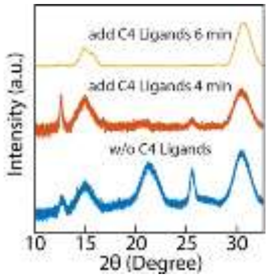

Figure S19. XRD patterns of C4 QDs. The C4 ligands (1 g) were added 2 minutes after injecting the Cs-precursor. After 4 minutes of annealing with C4 ligands, the (110) reflection is suppressed. After 6 minutes of annealing, the multi-layer diffraction fringe appears.

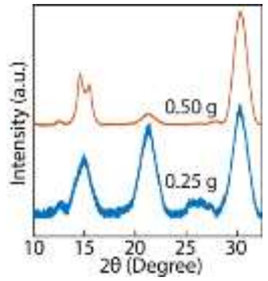

Figure S20. XRD of C4 QDs annealed with different amounts of C4 ligands.

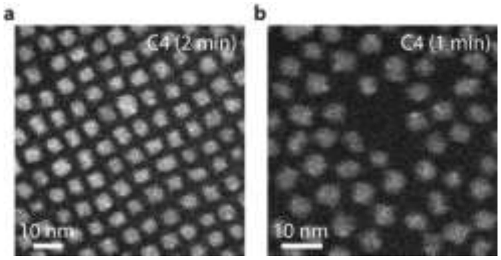

Figure S21. STEM images of QDs synthesized with (a) C4, extracted after 2 minutes of annealing, and QDs synthesized with (b) C6 extracted after 1 minute of annealing.

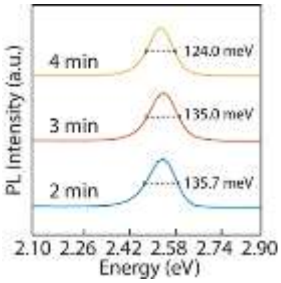

Figure S22. PL linewidths of PQDs annealed for 2, 3, and 4 minutes with 1.5g of C4 added.

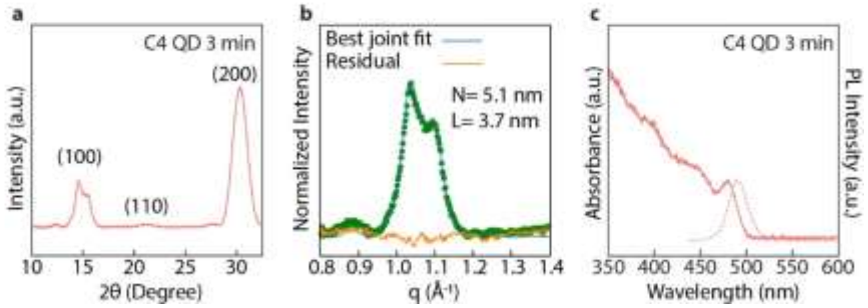

Figure S23. (a) XRD pattern, (b) multilayer diffraction fitting, and (c) absorption and PL spectra of a C4 QD sample.

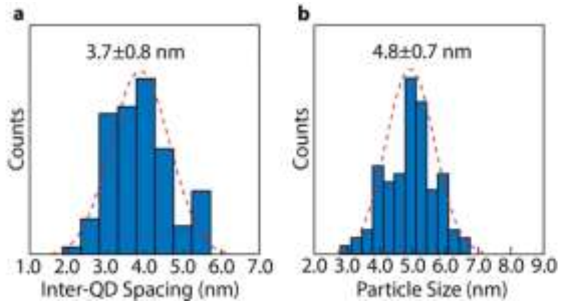

Figure S24. (a) Interparticle spacing and (b) size distribution histograms corresponding to QDs in Figure S23.

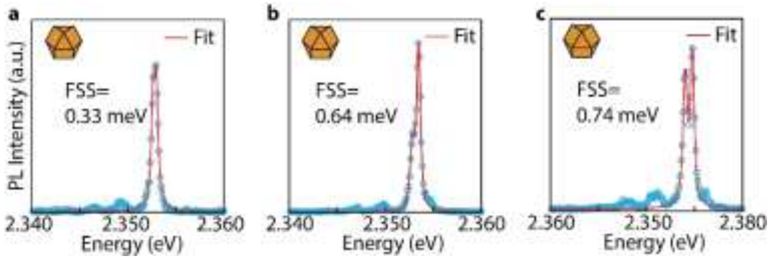

Figure S25. (a-c) Micro-PL spectra of additional polyhedral QDs at 3.2 K.

Supporting Information Note 3 Calculation of Ligand Coverage by quantitative ^{1}H NMR

DMSO-d$_6$ stock preparation

Stock solution of DMSO-d$_6$ spiked with mesitylene was prepared by adding 2.5 µL of mesitylene to 5 mL of DMSO-d$_6$ for a concentration of 3.6 mM.

Nanocrystal concentration estimation

C4 and C6 QDs were diluted by a factor of 100 into hexane and the absorption spectrum was recorded. Utilizing the size dependent optical cross section of $CsPbBr_3$ QDs and having determined the size of the ensembles from STEM imaging, the molar extinction coefficient for the QDs can be determined using the equation (eq. S5)

$$\sigma_\lambda = \varepsilon_\lambda \frac{2303}{N_A} \qquad (S5)$$

where (σ_λ) is the absorption cross section at a specific wavelength, (ε_λ) is the molar extinction coefficient with (N_A) being Avogadro's number. This allows for the concentration of the colloid to be determined via Beer's Law where (eq. S6),

$$A_\lambda = \varepsilon_\lambda b c_{QD} \qquad (S6)$$

Where (A_λ) is the absorbance at a given wavelength, (ε_λ) is the molar extinction coefficient at that wavelength, b is the pathlength, and (c_{QD}) is the concentration of the QD colloids.[7]

Ligand Fraction Estimation

QDs purified at least once with methyl acetate and capped with C4 or C6 ligands, dispersed in hexane were placed into an NMR tube and the hexane was removed under vacuum. To the dried QDs, 700 µL of DMSO-d6 spiked with mesitylene as an internal standard was added to digest the QDs and liberate any ligands bound to the surface of the QDs. The concentration of the diammonium ligands was determined by integrating the characteristic resonances corresponding the N-methyl group of C4 and C6 diammonium ligands relative to the aromatic protons of mesitylene at 6.76 ppm, while the oleylammonium concentration was determined by integrating the resonance of the vinyl protons at 5.32 ppm. The concentrations of the ligand species were calculated using the following equation (eq. S7):

$$\frac{In_x}{A_x} = \frac{3n_{IS}}{A_{IS}} \qquad (S7)$$

Where (I) is the number of protons corresponding to the peak being used for integration, (n_x) is the concentration of the ligand being quantified, (A_x) is the absolute integrated area of the peak of the ligand being quantified, (n_{IS}) is the concentration of the internal standard used mesitylene, and (A_{IS}) is the absolute integrated area of mesitylene.

We calculate the availability of binding sites by assuming the particles are a perfect cube using the theoretical binding site density of (2.9 nm^{-1})[27] and calculated the surface area of a perfect cube using the average size of the QDs from STEM imaging. From this we divide the concentration of the ligand observed in the digested sample by the total concentration of binding sites available in the sample based on the previously determined QD concentration to determine

the ligand coverage. In the case of the Oleylammonium, we treat it as 67% bound as determined from ^{1}H NMR DOSY measurements as seen in Figure S14. The dicationic ligand concentration was multiplied by two to represent the bidentate nature of its binding when determining the coverage. This is represented by the equation (eq. S8)

$$Ligand\ Coverage = \frac{[Oleylammonium] * 0.67 + [Dicationic\ Ligand] * 2}{[Binding\ Sites]} \qquad (S8)$$

For the values shown in Table S3 we have calculated the amount of oleylammonium ligands using the α-CH_2 protons of OAm^+ (~2.75 ppm), whose signals are contributed solely by OAm^+. The oleate content was determined by taking the difference in the concentration of the oleyl ligands determined by integrating the vinyl peak at 5.32 ppm and the concentration of OAm^+ determined by integration of the α-CH_2 of OAm^+ at ~2.75 ppm. As listed in Table S5, oleic acid/oleate ligands have a much lower concentration than that of the oleylamine/oleylammonium ligands.

Table S5

Estimated ligand concentrations of $CsPbBr_3$ QDs treated with C4 and C6

Concentration	C4	C6
Mesitylene	3.6×10^{-3} M	3.6×10^{-3} M
Binding Sites (Theoretical)	5.1×10^{-3} M	8.9×10^{-3} M
Dicationic Ligands	1.4×10^{-4} M	7.2×10^{-4} M
Oleylammonium Ligands	1.1×10^{-3} M	5.1×10^{-3} M

References

(1) Atteberry, M. L.; Mi, C.; Chandra, S.; Hidayatova, L.; Dong, Y. Unraveling the Growth Mechanism of Strongly Confined $CsPbBr_3$ Perovskite Quantum Dots under Thermodynamic Equilibrium Control. *Chem. Mater.* **2024**, *36* (9), 4521-4529

(2) Protesescu, L.; Yakunin, S.; Bodnarchuk, M. I.; Krieg, F.; Caputo, R.; Hendon, C. H.; Yang, R. X.; Walsh, A.; Kovalenko, M. V. Nanocrystals of Cesium Lead Halide Perovskites ($CsPbX_3$, X = Cl, Br, and I): Novel Optoelectronic Materials Showing Bright Emission with Wide Color Gamut. *Nano Lett.* **2015**, *15* (6), 3692-3696

(3) Ginterseder, M.; Sun, W.; Shcherbakov-Wu, W.; McIsaac, A. R.; Berkinsky, D. B.; Kaplan, A. E. K.; Wang, L.; Krajewska, C.; Šverko, T.; Perkinson, C. F.; et al. Lead Halide Perovskite Nanocrystals with Low Inhomogeneous Broadening and High Coherent Fraction through Dicationic Ligand Engineering. *Nano Lett.* **2023**, *23* (4), 1128-1134

(4) Schindelin, J.; Arganda-Carreras, I.; Frise, E.; Kaynig, V.; Longair, M.; Pietzsch, T.; Preibisch, S.; Rueden, C.; Saalfeld, S.; Schmid, B.; et al. Fiji: an open-source platform for biological-image analysis. *Nat. Methods* **2012**, *9* (7), 676-682

(5) Arganda-Carreras, I.; Kaynig, V.; Rueden, C.; Eliceiri, K. W.; Schindelin, J.; Cardona, A.; Sebastian Seung, H. Trainable Weka Segmentation: a machine learning tool for microscopy pixel classification. *Bioinformatics* **2017**, *33* (15), 2424-2426

(6) Mi, C.; Gee, G. C.; Lander, C. W.; Shin, D.; Atteberry, M. L.; Akhmedov, N. G.; Hidayatova, L.; DiCenso, J. D.; Yip, W. T.; Chen, B.; et al. Towards non-blinking and photostable perovskite quantum dots. *Nat. Commun.* **2025**, *16* (1), 204

(7) Puthenpurayil, J.; Cheng, O. H.-C.; Qiao, T.; Rossi, D.; Son, D. H. On the determination of absorption cross section of colloidal lead halide perovskite quantum dots. *J. Chem. Phys.* **2019**, *151* (15),

(8) De Roo, J.; Ibáñez, M.; Geiregat, P.; Nedelcu, G.; Walravens, W.; Maes, J.; Martins, J. C.; Van Driessche, I.; Kovalenko, M. V.; Hens, Z. Highly Dynamic Ligand Binding and Light Absorption Coefficient of Cesium Lead Bromide Perovskite Nanocrystals. *ACS Nano* **2016**, *10* (2), 2071-2081

(9) Imran, M.; Ijaz, P.; Goldoni, L.; Maggioni, D.; Petralanda, U.; Prato, M.; Almeida, G.; Infante, I.; Manna, L. Simultaneous Cationic and Anionic Ligand Exchange For Colloidally Stable CsPbBr3 Nanocrystals. *ACS Energy Letters* **2019**, *4* (4), 819-824

(10) Berezovska, Y.; Sabisch, S.; Bernasconi, C.; Sahin, Y.; Bertolotti, F.; Guagliardi, A.; Bodnarchuk, M. I.; Dirin, D. N.; Kovalenko, M. V. Tightly yet Dynamically Bound Aliphatic Guanidinium Ligands for Lead Halide Perovskite Nanocrystals. *J. Am. Chem. Soc.* **2025**, *147* (39), 35446-35455

(11) Sakata, M.; Nishiwaki, T.; Harada, J. Neutron Diffraction Study of the Structure of Cubic $CsPbBr_3$. *J. Phys. Soc. Jpn.* **1979**, *47* (1), 232-233

(12) Hjorth Larsen, A.; Jørgen Mortensen, J.; Blomqvist, J.; Castelli, I. E.; Christensen, R.; Dułak, M.; Friis, J.; Groves, M. N.; Hammer, B.; Hargus, C.; et al. The atomic simulation environment—a Python library for working with atoms. *J. Phys. Condens. Matter.* **2017**, *29* (27), 273002

(13) Kühne, T. D.; Iannuzzi, M.; Del Ben, M.; Rybkin, V. V.; Seewald, P.; Stein, F.; Laino, T.; Khaliullin, R. Z.; Schütt, O.; Schiffmann, F.; et al. CP2K: An electronic structure and molecular dynamics

software package - Quickstep: Efficient and accurate electronic structure calculations. *J. Chem. Phys.* **2020**, *152* (19),
(14) Perdew, J. P.; Burke, K.; Ernzerhof, M. Generalized Gradient Approximation Made Simple. *Phys. Rev. Lett.* **1996**, *77* (18), 3865-3868
(15) Lippert, B. G.; Parrinello, J. H.; Michele. A hybrid Gaussian and plane wave density functional scheme. *Mol. Phys.* **1997**, *92* (3), 477-488
(16) VandeVondele, J.; Hutter, J. Gaussian basis sets for accurate calculations on molecular systems in gas and condensed phases. *J. Chem. Phys.* **2007**, *127* (11), 114105
(17) Goedecker, S.; Teter, M.; Hutter, J. Separable dual-space Gaussian pseudopotentials. *Phys. Rev. B.* **1996**, *54* (3), 1703-1710
(18) Hartwigsen, C.; Goedecker, S.; Hutter, J. Relativistic separable dual-space Gaussian pseudopotentials from H to Rn. *Phys. Rev. B.* **1998**, *58* (7), 3641-3662
(19) Krack, M. Pseudopotentials for H to Kr optimized for gradient-corrected exchange-correlation functionals. *Theor. Chem. Acc.* **2005**, *114* (1), 145-152
(20) Grimme, S.; Antony, J.; Ehrlich, S.; Krieg, H. A consistent and accurate ab initio parametrization of density functional dispersion correction (DFT-D) for the 94 elements H-Pu. *J. Chem. Phys.* **2010**, *132* (15),
(21) Grimme, S.; Ehrlich, S.; Goerigk, L. Effect of the damping function in dispersion corrected density functional theory. *J. Comput. Chem.* **2011**, *32* (7), 1456-1465
(22) VandeVondele, J.; Krack, M.; Mohamed, F.; Parrinello, M.; Chassaing, T.; Hutter, J. Quickstep: Fast and accurate density functional calculations using a mixed Gaussian and plane waves approach. *Comput. Phys. Commun.* **2005**, *167* (2), 103-128
(23) VandeVondele, J.; Hutter, J. An efficient orbital transformation method for electronic structure calculations. *J. Chem. Phys.* **2003**, *118* (10), 4365-4369
(24) Kolafa, J. Time-reversible always stable predictor–corrector method for molecular dynamics of polarizable molecules. *J. Comput. Chem.* **2004**, *25* (3), 335-342
(25) Frigo, M.; Johnson, S. G. The Design and Implementation of FFTW3. *Proc. IEEE* **2005**, *93* (2), 216-231
(26) Humphrey, W.; Dalke, A.; Schulten, K. VMD: Visual molecular dynamics. *Journal of Molecular Graphics* **1996**, *14* (1), 33-38
(27) Stelmakh, A.; Aebli, M.; Baumketner, A.; Kovalenko, M. V. On the Mechanism of Alkylammonium Ligands Binding to the Surface of $CsPbBr_3$ Nanocrystals. *Chem. Mater.* **2021**, *33* (15), 5962-5973
